# Oncogenic function and clinical implications of SLC3A2-NRG1 fusion in invasive mucinous adenocarcinoma of the lung

**DOI:** 10.18632/oncotarget.11913

**Published:** 2016-09-08

**Authors:** Dong Hoon Shin, Donghoon Lee, Dong Wan Hong, Seung Hyun Hong, Jung-Ah Hwang, Byung Il Lee, Hye Jin You, Geon Kook Lee, In-Hoo Kim, Yeon-Su Lee, Ji-Youn Han

**Affiliations:** ^1^ Lung Cancer Branch, Graduate School of Cancer Science and Policy, National Cancer Center, Ilsandong-gu, Goyang-si, Gyeonggi-do, Republic of Korea; ^2^ Cancer Genomic Branch, Graduate School of Cancer Science and Policy, National Cancer Center, Ilsandong-gu, Goyang-si, Gyeonggi-do, Republic of Korea; ^3^ Biomolecular Function Research Branch, Graduate School of Cancer Science and Policy, National Cancer Center, Ilsandong-gu, Goyang-si, Gyeonggi-do, Republic of Korea; ^4^ Cancer Cell and Molecular Biology Branch, Research Institute and Hospital, Graduate School of Cancer Science and Policy, National Cancer Center, Ilsandong-gu, Goyang-si, Gyeonggi-do, Republic of Korea; ^5^ Department of System Cancer Science, Graduate School of Cancer Science and Policy, National Cancer Center, Ilsandong-gu, Goyang-si, Gyeonggi-do, Republic of Korea

**Keywords:** SLC3A2-NRG1, invasive mucinous adenocarcinoma (IMA), ERBB2, ERBB3

## Abstract

The neuregulin 1 (NRG1) fusion is a recently identified novel driver oncogene in invasive mucinous adenocarcinoma of the lung (IMA). After identification of a case of SLC3A2-NRG1 in a patient with IMA, we verified this fusion gene in a cohort of 59 patients with IMA. Targeted cancer panel sequencing and RT-PCR identified the possible coexistence of other driver oncogenes. Among 59 IMAs, we found 16 NRG1 fusions (13 SLC3A2-NRG1 and 3 CD74-NRG1). Of 16 patients with NRG1 fusions, concurrent KRAS codon 12 mutations were found in 10 cases. We also found concurrent NRAS Q61L mutation and EML4-ALK fusion in additional two cases with NRG1 fusions. When comparing overall survival (OS) according to the presence of NRG1 fusions showed that patients harboring NRG1 fusions had significantly inferior OS than those without NRG1 fusions (hazard ratio = 0.286; 95% confidence interval, .094 to .865). Ectopic expression of the SLC3A2-NRG1 fusion in lung cancer cells increased cell migration, proliferation and tumor growth *in vitro* and in xenograft models, suggesting oncogenic function for the fusion protein. We found that the SLC3A2-NRG1 fusion promoted ERBB2-ERBB3 phosphorylation and heteroduplex formation and activated the downstream PI3K/AKT/mTOR pathway through paracrine signaling. These findings suggested that the SLC3A2-NRG1 fusion was a driver in IMA with an important prognostic impact. SLC3A2-NRG1 should be considered a therapeutic target for patients with IMA.

## INTRODUCTION

Lung cancer is the leading cause of cancer-related death worldwide and is highly heterogeneous at the molecular level [1]. Adenocarcinoma is now the most common histological subtype of lung cancer, and subclassification is clinically important for deciding the best course of treatment [2]. To date, tumor genotype analysis has identified driver alterations in 60-80% of lung adenocarcinoma patients according to ethnicity and smoking status [3]. The National Cancer Institute's Lung Cancer Mutation Consortium tested 1007 patients with lung adenocarcinoma and detected driver mutations in 62%. Specifically, 24% of patients had KRAS mutations, 17% had EGFR mutations, 8% had ALK fusions, 4% had other EGFR mutations, 2% had ERBB2 mutations, 2% had BRAF mutations, 0.7% had PIK3CA mutations, 0.6% had MET amplification, 0.5% had NRAS mutations, and 0.2% had MEK1 mutations [4]. Most mutations are mutually exclusive and associated with sensitivity or resistance to specific targeted therapies. In particular, EGFR, ALK and ROS1 mutations are treatable with kinase inhibitors and are more common in never-smokers with lung adenocarcinoma [5–7]. Many clinical studies show that genotype-based targeted therapies result in significant improvements in response rate, progression-free survival and quality of life compared with conventional chemotherapies [8–13]. Genotype-based approaches have contributed to a paradigm shift in the treatment of lung cancer. Tumor genotyping at disease presentation is currently used to select among available targeted therapies for patients with lung adenocarcinoma. However, approximately 20-40% of lung adenocarcinomas lack a known driver mutation. Thus, novel driver oncogene research is an active area of investigation.

To identify novel driver oncogenes, we previously examined oncogenic alterations in 48 surgically resected lung adenocarcinomas from Korean never-smokers [14]. Using conventional methods, we found driver oncogenic alterations in 36 patients; of these, 25 were EGFR mutations, 4 KRAS mutations, 3 ALK fusions, 2 ROS1 fusions, and 2 RET fusions. One patient had concurrent EGFR L858R and PIK3CA mutations. We performed targeted next-generation sequencing (NGS) on 12 tumor samples that did not contain known genetic alterations identifiable using conventional assays. We found additional EGFR, KRAS and PIK3CA mutations in 5 of 12 samples through targeted NGS. After excluding all samples with known mutations, we performed NGS RNA sequencing on 7 tumor samples without known driver mutations. This process identified a fusion gene, SLC3A2-NRG1, in a patient with invasive mucinous adenocarcinoma (IMA).

IMA is a unique histological variant of lung cancer and accounts for approximately 5% of lung adenocarcinoma. IMA is most strongly correlated with the KRAS mutation and usually shows poor response to chemotherapy [16, 17]. Recent advances in comprehensive molecular studies identified novel drivers including CD74-NRG1 fusions in IMA [18, 19]. Although recent studies showed that NRG1 fusions may exist in never-smokers and KRAS-wild type tumors exclusively, because of small sample sizes, correlation with other genomic alterations and clinical impact in IMAs has not been fully evaluated. We report the prevalence of NRG1 fusions in a cohort of patients with IMA and association with other genetic alterations. Further, we investigated the oncogenic functions, pathways and the clinical implications of the SCL3A2-NRG1 fusion in IMA.

## RESULTS

### Identification and validation of NRG1 fusions in invasive mucinous adenocarcinoma of the lung

**Figure 1:**
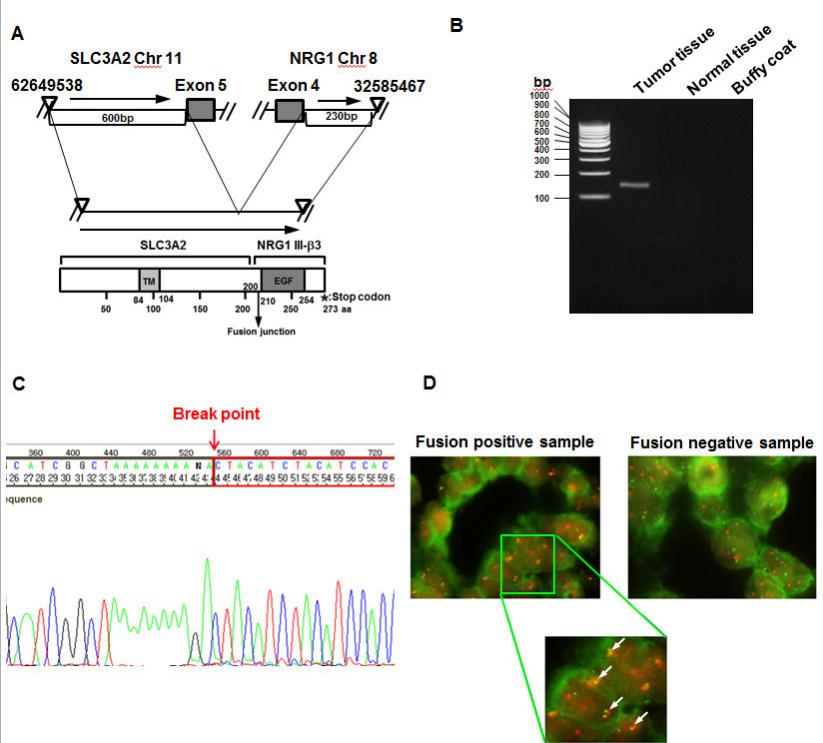
Identification of an SLC3A2-NRG1 fusion in lung mucinous adenocarcinoma **A.**, SLC3A2 gene mapped to chromosome 11 and the NRG1 gene mapped to chromosome 8, with the same orientation. SLC3A2 was disrupted at ~600 bp upstream of exon 5 and NRG1 at ~230 bp downstream of exon 4 to generate the SLC3A2-NRG1 fusion gene. Schematic representation of the TM (transmembrane domain), fusion junction, EGF, and stop codon of SLC3A2-NRG1. **B.**, Detection of gene-fusion transcripts by RT-PCR in tumor and normal tissue. **C.**, Identification of fusion gene and breakpoint using direct sequencing. **D.**, The SLC3A2-NRG1 fusion product was detected by Florescence *In Situ* Hybridization (FISH). Cancer and normal FFPE tissue samples of IMA patients harboring the SCL3A2-NRG1 fusion product were subjected to FISH analysis. Customized MacProbes for SLC3A2 (NCC-HJA-A, red) and NRG1 (NCC-HJA-B, green) were used. Magnification is 1:1000 for upper panel and 1:2,000 for lower panel. Arrows show break-apart signals of the fusion gene SLC3A2-NRG1 (yellow or orange).

We performed RNA sequencing of surgically resected IMAs of the lung using NGS technology and identified a fusion SLC3A2-NRG1. In the SLC3A2-NRG1 fusion, the first five exons of SLC3A2 appeared to be fused with exon 4 of NRG1 through the end of the coding sequence (Figure 1A). The fusion transcript in the tumor sample was verified using three methods: RT-PCR (Figure 1B), direct sequencing (Figure 1C) and FISH (Figure 1D). To investigate the frequency of SLC3A2-NRG1 fusion in IMA, we tested an additional 59 IMA samples obtained from patients who underwent curative surgical resection, identifying 13 SLC3A2-NRG1 fusions (27% frequency). To evaluate coexistence with other fusions, we tested known fusion genes CD74-NRG1, TPM3-ROS1, SDC4-ROS1, SLC34A2-ROS1, CD74-ROS1, EZR-ROS1, LRIG-ROS1, KIF5B-RET, CCDC6-RET1, EZR-ERBB4, TRIM24-BRAF, KIAA1468-RET, EML4-ALK, and KIF5B-ALK. Of 59 IMA samples tested, 16 NGR1 fusions (13 SLC3A2-NRG1 and 3 CD74-NRG1) and 3 EML4-ALK fusions were identified using RT-PCR and direct sequencing (Summarized in Supplemental Figure S1 and Supplemental Table S1). To characterize IMAs with NRG1 fusions, we compared clinicopathological features of IMAs according to the presence of an NRG1 fusion (Table 1). The 16 patients with tumors harboring NRG1 fusions had a median age of 64 years (range 36-84 years), and the majority (12 of 16, 75%) had pathological stage I disease. Seven (44%) were women, and eight (50%) were never-smokers. No significant differences in clinicopathological features were observed between NRG1 fusion-positive and fusion-negative IMAs. Although we discovered the SLC3A2-NRG1 fusion in a pan-negative lung adenocarcinoma detected by conventional Sanger sequencing, we performed targeted cancer panel deep sequencing to identify possible coexistence with known mutations. We tested 739 mutations in 46 key cancer genes (see gene list in methods). Variants with a minimum coverage of 500 reads containing at least 20 mutant reads were selected. Among the 16 patient samples with NRG1 fusions, concurrent KRAS codon-12 mutations were found in 9. We also found concurrent NRAS Q61L mutations and EML4-ALK fusions in additional 2 with NRG1 fusions. A summary of driver mutations in 59 IMA samples is in Supplemental Figure S2 and Supplemental Table S2.

**Table 1:** Clinical characteristics of lung mucinous adenocarcinoma according to NRG1 fusion (*N*=59)

		NRG1 fusion positive (%)	NRG1 fusion negative (%)	*P*
**N**		**16 (27)**	**43 (73)**	
**Age**	**Median (range)**	**64 (36-84)**	**65 (41-79)**	
**Smoking**	**Never** **Ever**	**8 (50)** **8 (50)**	**27 (63)** **16 (37)**	**.374**
**Sex**	**Male** **Female**	**9 (56)** **7 (44)**	**18 (42)** **25 (58)**	**.324**
**Stage**	**I** **II** **III**	**12 (75)** **3 (19)** **1 (6)**	**33 (77)** **5 (12)** **5 (12)**	**.680**

### Oncogenic function of the SLC3A2-NRG1 fusion in non-small cell lung cancer

**Figure 2:**
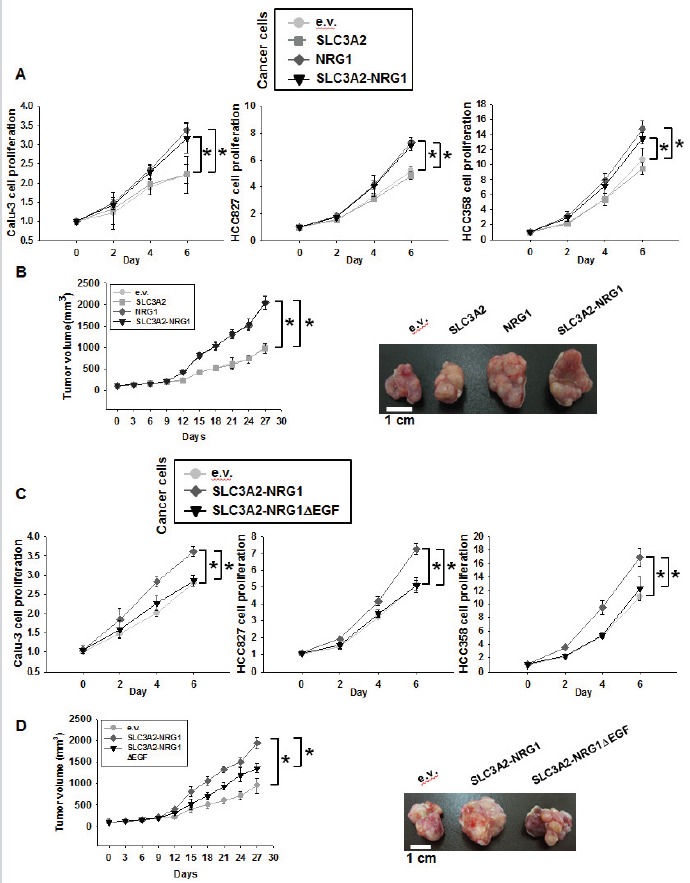
Oncogenic effects of expression of SLC3A2-NRG1 in cancer cells **A.** and **C.**, Expression vectors for empty vector (e.v.), SLC3A2, NRG1, SLC3A2-NRG1 and SLC3A2-NRG1Δ EGF transfected into Calu-3, HCC827, and HCC358. Cell proliferation was determined using MTT assays every 2 days for 6 days. Student's *t*-test, average ± SD; *n* = 6, * *p < 0.05*. **B.** and **D.**, HCC358 cells were infected with retrovirus expressing e.v., SLC3A2, NRG1, SLC3A2-NRG1, or SLC3A2-NRG1Δ EGF. Infected cells were selected by puromycin (2 μg/ml) and injected subcutaneously into nude mice. Tumor formation was examined for 27 days. Student's *t*-test, average ± SEM; *n* = 5, * *p < 0.05*.

We visualized the potential fusion transcript based on information from RNA sequencing and protein annotation (Supplemental Figure S3). SLC3A2-NRG1 fusion proteins were composed of an SLC3A2 transmembrane domain and NRG1 cytosolic domain (NRG1 type III-β3 isoform) with an EGF-like domain. NRG1 type III generates a membrane-tethered N-terminal fragment known to mediate juxtacrine signaling through ERBB2 and ERBB3 receptors [20]. To further study function, we screened 11 NSCLC cell lines and one human bronchial epithelial cell line to quantify ERBB1 and ERBB4 expression (Supplemental Figure S4). Three cancer cells (black color, Calu-3, HCC827, and HCC358) showed higher ERBB2 and ERBB3 levels than other cell lines and were selected for further studies. To examine the function of the SLC3A2-NRG1 fusion gene in cancer cells, SLC3A2, NRG1, and the fusion gene were overexpressed in Calu-3, HCC827, and HCC358 by transient transfection. Overexpression was verified by immunoblotting and RT-PCR in HEK 293T cells (Supplemental Figure S5). Tumor xenografts in nude mice were generated for measuring tumor volume and weight. Proliferation, and tumor volume and weight were analyzed for cancer cells ectopically expressing SLC3A2, NRG1 and SLC3A2-NRG1 (Figure 2A, 2B, and Supplemental Figure S6A). Compared with empty-vector control (e.v.) and SLC3A2, cancer cells expressing NRG1 and SLC3A2-NRG1 fusion genes showed substantial enhancement. To confirm the oncogenic function of the NRG1 part of the fusions, a truncated version of the fusion lacking the EGF-like domain (SLC3A2-NRG1ΔEGF) was made. Truncation was verified by western blots and ELISA for the EGF-like domain (Supplemental Figure S5A and S7). Increased cell proliferation, tumor volume and weight with the SLC3A2-NRG1 fusion were significantly eliminated when a SLC3A2-NRG1Δ EGF truncated form was used in the same cancer cells (Figure 2C, 2D, and Supplemental Figure S6B). These results suggested that the part of NRG1 with the EGF-like domain in the SLC3A2-NRG1 fusion protein was critical for NSCLC proliferation and tumorigenesis.

**Figure 3:**
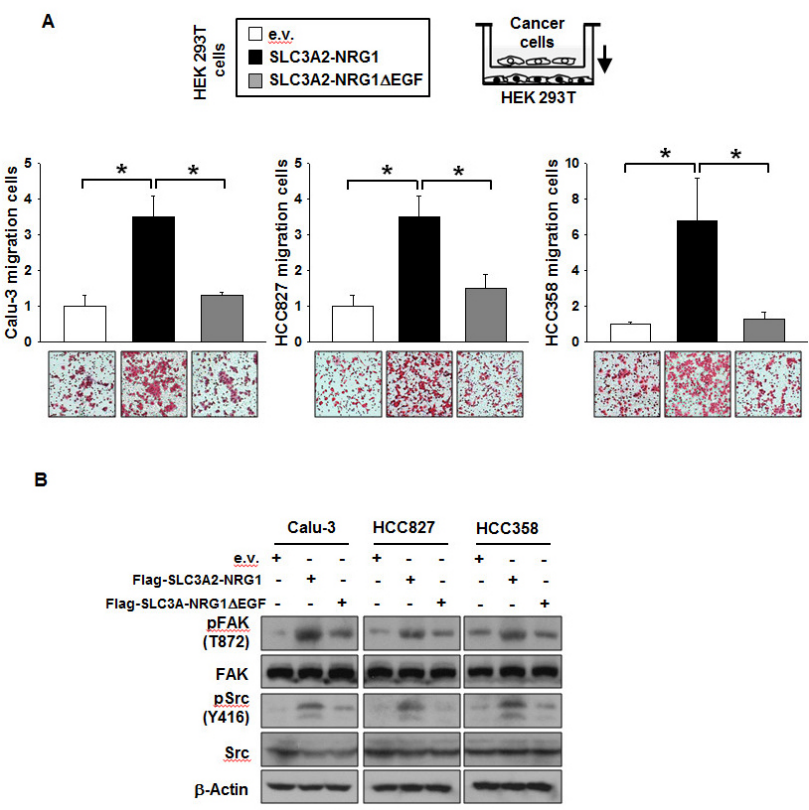
SLC3A2-NRG1 fusion gene increases cancer cell migration **A.**, HEK 293T cells transfected with e.v., SLC3A2-NRG1 or SLC3A2-NRG1Δ EGF were co-cultured in Boyden chambers (lower, HEK 293T; upper, Calu-3, HCC827 or HCC358). Cells were incubated for 24 h and stained and counted per 3.8 cm^2^. Student's *t*-test, average ± SD; *n* = 3, * *p < 0.05*. **B.**, Migration related to signaling with FAK and Src downstream of ERBB2-ERBB3.

To examine whether the SLC3A2-NRG1 fusion protein enhanced cancer cell migration, migration assays were performed with Boyden chambers. Cancer cells expressing SLC3A2-NRG1 in the chamber with HEK 293T cells migrated significantly more than cells with empty vector. However, cell migration was not increased by SLC3A2-NRG1Δ EGF expression (Figure 3A). Cancer cell migration induced by the SLC3A2-NRG1 fusion protein was due to an increase in pFAK and pSrc by the SLC3A2-NRG1 fusion protein; this was not induced by SLC3A2-NRG1Δ EGF (Figure 3B). These results indicated that the EGF domain in the NRG1 part of the SLC3A2-NRG1 fusion augmented cell proliferation and migration.

### Oncogenic function of the SLC3A2-NRG1 fusion through paracrine signaling

**Figure 4:**
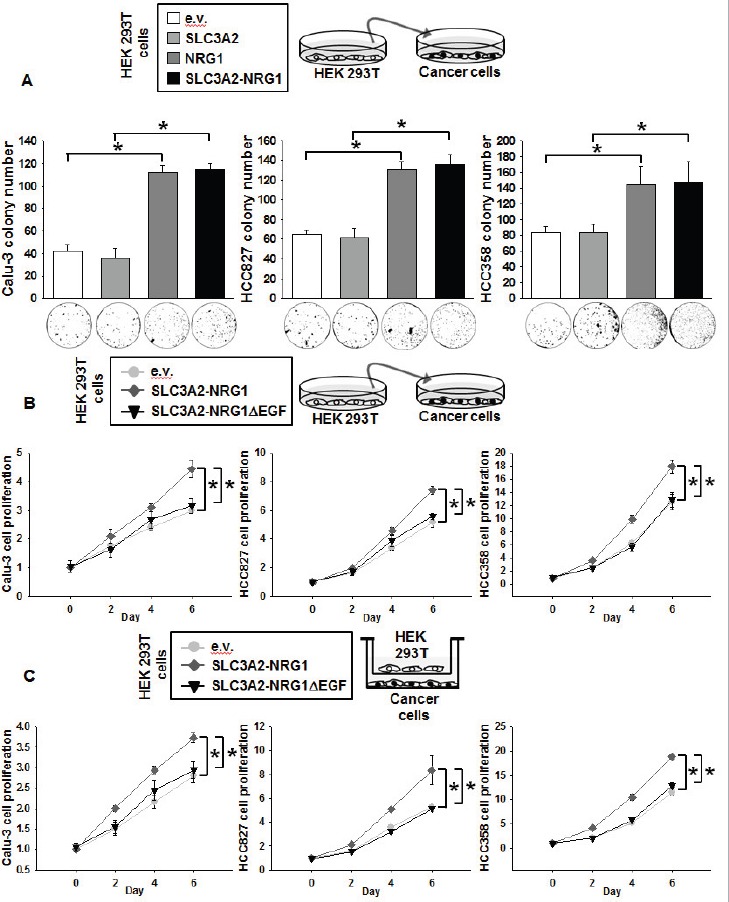
NRG1 secreted from SLC3A2-NRG1 fusion gene stimulates cancer cell growth **A.**, HEK 293T cells transfected with e.v., SLC3A2, NRG1, SLC3A2-NRG1, and/or SLC3A2-NRG1Δ EGF. Cancer cells were seeded with 0.4% top agar and cultured with conditioned medium from HEK 293T cells and fresh medium for 28 days. Cell colonies were visualized using crystal violet and counted per 3.8 cm^2^. Student's *t*-test, average ± SD; *n* = 3, * *p < 0.05*. **B.**, HEK 293T and cancer cells were transfected as described in A. Cancer cells were cultured in 1:1 v:v conditioned medium from HEK 293T cells and fresh medium. Cell growth was analyzed by MTT assays. Student's *t*-test, average ± SD; *n* = 6, * *p < 0.05*. **C.**, HEK 293T cells transfected as in A and co-cultured in Boyden chambers (upper, HEK 293T; lower, Calu-3, HCC827, or HCC358). Cell proliferation was measured by MTT assays. Student's *t*- test, average ± SD; *n* = 6, * *p < 0.05*.

Like NRG1 types I and II, NRG1 type III-β3 is released by cleavage by proteases in families such as ADAM (a disintegrin and metalloproteinase) [20]. Hence, we hypothesized that a soluble protein from SLC3A2-NRG1 fusion protein could be released to influence the oncology of cancer cells. To examine this hypothesis, we applied conditioned medium from HEK 293T cells to cancer cells. As in the co-culture experiments, colony formation in HEK 293T conditioned media depended on SLC3A2-NRG1 and NRG1 expression in the HEK 293T cells (Figure 4A). Cancer cell growth was also facilitated by medium from SLC3A2-NRG1-overexpressing HEK 293T cells but was not affected by medium from SLC3A2-NRG1Δ EGF HEK 293T cells (Figure 4B). HEK 293T cells expressing e.v., SLC3A2-NRG1 or SLC3A2-NRG1Δ EGF plasmids were co-cultured with cancer cells in a Boyden chamber. Cancer cell proliferation was affected by overexpression of SLC3A2-NRG1 in the HEK 293T cells, but not by expression of SLC3A2-NRG1Δ EGF (Figure 4C). These results supported that a soluble protein from the SLC3A2-NRG1 fusion is a cancer cell proliferating factor.

### Oncogenic signaling of the SLC3A2-NRG1 fusion through ERBB2-ERBB3 heterocomplex

**Figure 5:**
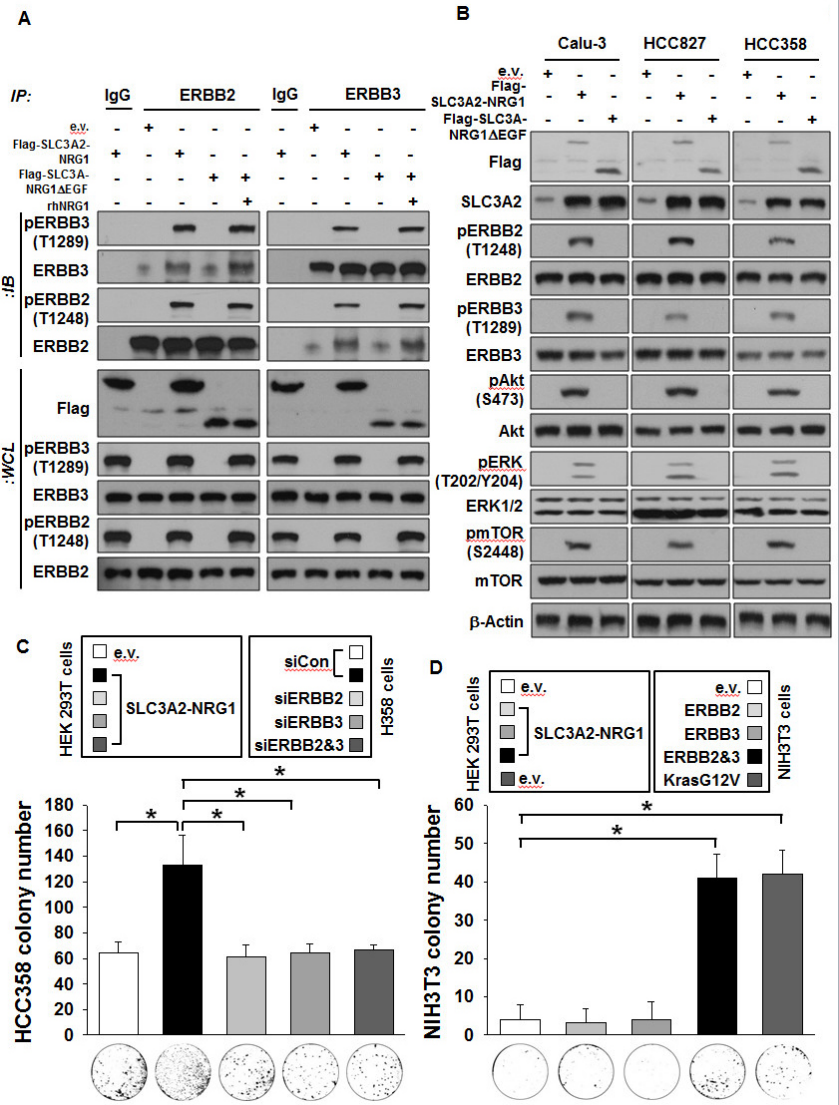
SLC3A2-NRG1 fusion gene induces ERBB2-ERBB3 heterocomplex formation and downstream signaling **A.**, HCC358 transfected and treated with 10 ng/ml recombinant human NRG1-β1 EGF domain (rhNRG1-β1-EGF) as positive control. Cell lysates were subjected to IP and IB. **B.**, Cancer cells were transfected with indicated plasmids and measured for ERBB2, ERBB3, and downstream signaling. **C.**, HCC 358 cells were transfected with specific ERBB2 and/or ERBB3 siRNA (80 nM) and transferred to soft agar. Cells in 0.4% top agar were placed on 1% bottom agar. Media containing e.v. and SLC3A2-NRG1 was replaced every 3 days for 3 weeks. Cells were stained with crystal violet (lower) and counted (upper). Bars represent mean + SD (*n* = 4; * *p < 0.*05). **D.**, NIH3T3 cells were transfected with ERBB2 and/or ERBB3 plasmids (3 μg/μl) or KrasG12V (3 μg/μl) as a positive control and transferred to soft agar. Soft agar medium was changed as in C. Bars represents mean + SD (*n* = 4; * *p <* 0*.*05).

The ERBB2-ERBB3 heterocomplex induced by NRG1 is emerging as important for growth of non-small cell lung, breast, and melanoma cell growth [21–23]. We tested if the SLC3A2-NRG1 fusion also enhanced the ERBB2-ERBB3 complex. Co-immunoprecipitation showed that, compared with e.v., expression of the ectopic SLC3A2-NRG1 fusion gene increased phosphorylation of the two receptors when combined with treatment with a recombinant human NRG1-EGF domain as a positive control (Figure 5A). Also, downstream signaling from AKT, ERK, and mTOR and their phosphorylation increased; this was substantially abrogated by SLC3A2-NRG1Δ EGF (Figure 5B). To confirm these results, we performed colony formation assays using loss-of-function or gain-of-function versions of ERBB2 and ERBB3. HCC358 colonies with media from HEK 293T cells expressing SLC3A2-NRG1 were not formed with knockdown ERBB2 and/or ERBB3 (Figure 5C). In contrast, colony formation of NIH3T3, ERBB2 and ERBB3 null cells occurred with all expressed forms of ERBB2 and ERBB3 (Figure 5D). The effect of siRNA and plasmid expression of ERBB2 and ERBB3 was verified by Western blots (Supplemental Figure S8). The results suggested that the SLC3A2-NRG1 fusion activated ERBB2-ERBB3 heterocomplex signaling through a juxtacrine and/or autocrine mechanism.

### Clinical outcomes of IMA with or without NRG1 fusions

**Figure 6:**
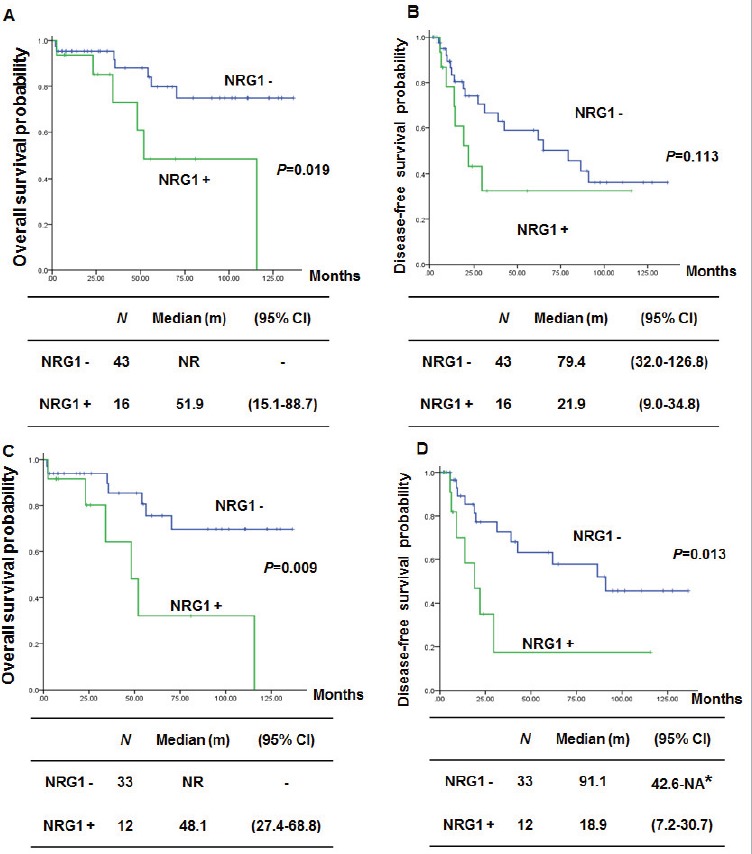
Comparison of survival according to NRG1 fusion in lung mucinous adenocarcinoma **A.** and **B.**, Overall survival (OS) and disease-free survival (DFS) of NRG1-negative and NRG1-positive patients. **C.** and **D.**, OS and DFS of patients with stage I disease. NR: Not reached. NA: Not attained. * the upper level of 95% CI was not attained due to the small number of events.

We compared survival according to the presence of the NRG1 fusion. Patients harboring tumors with NRG1 fusions showed inferior overall survival (OS) compared with those without NRG1 fusions (Figure 6A, *P* = 0.019). Patients harboring tumors with NRG1 fusions also showed a trend towards shorter disease-free survival (DFS) compared with those without NRG1 fusion (Figure 6B). To exclude the impact of stage on survival, we compared OS and DFS only in patients with stage I disease. Patients with NRG1 fusions showed significantly inferior OS and DFS compared to those without NRG1 fusions (Figure 6C and 6D, *P* = 0.009 and 0.013).

## DISCUSSION

NRG1 fusions are found in a novel molecular subset of lung adenocarcinomas with distinct mucinous features. Since a CD74-NRG1 fusion was identified in 2014, other groups reported this fusion in patients with IMA. The first group found CD74-NRG1 in never-smoker women with IMA at a frequency of 27% (4/15) [24]. A group in Taiwan also found the CD74-NRG1 fusion in 1 of 13 patients with IMA with frequency of 8% [18]. A Japanese group identified a new SLC3A2-NRG1 fusion in addition to the CD74-NRG1 fusion in a subset of patients with IMA. They found NRG1 fusions in smokers as well as non-smokers in IMA at a frequency of 17.6% (6/34) [25]. VAMP2-NRG1 was reported in a 67-year-old never-smoker woman with adenocarcinoma with no mutation in EGFR, KRAS and BRAF and with no fusion genes involving ALK, ROS1 or RET [26]. These fusion genes are expected to have an oncological function because part of CD74, SLC3A or VAMP2 is predicted to replace the transmembrane domain of wild-type NRG1 type III-β3, which contains a membrane-tethered EGF-like domain. The EGF-like domain in the NRG1 fusion is expected to produce oncogenic signals through ERBB2-ERBB3. The function of only the CD74-NRG1 fusion was discovered experimentally; the rest remains hypothesized.

Our study discovered an SLC3A2-NRG1 fusion in an IMA from a never-smoker and confirmed the frequency of the SLC3A2-NRG1 fusion in a cohort of patients with IMA as 27% (16/59), irrespective of smoking status. Despite examining a relatively higher number of IMA cases, we did not find any clinical features associated with the NRG1 fusion except for the mucinous histological type. All instances of NRG1 fusions reported thus far have been found only in East Asian populations. The Taiwan group detected no CD74-NRG1 fusions in 109 adenocarcinomas of subtypes other than IMA [18]. These findings suggest that NRG1 fusions may occur frequently in East Asian populations with IMA.

IMAs primarily contain KRAS but not EGFR mutations [16]. We examined the mutational status of 59 cases of IMA using Ion Torrent. KRAS mutations were the most common (29/59) followed by EGFR (3/59) and NRAS (1/59) mutations. Using RT-PCR, we analyzed other fusions involving ALK, RET, ROS1, BRAF, and ERBB4. We found additional EML4-ALK fusions in two IMAs. Two previous studies reported NRG1 fusions that were mutually exclusive with KRAS mutations [24, 25]. Unlike these reports, we found that NRG1 fusions frequently occur with other mutations, especially KRAS. Among 16 samples with NRG1 fusions, 10 had concurrent KRAS mutations (5G12D, 4G12V, 1G12C) and one had a concurrent NRAS Q16L mutation. Since previous studies analyzed only a limited number of IMA samples and we analyzed a larger number, NRG1 fusions with other mutations might not be common but also could be excluded. As the most common oncogenic event in lung adenocarcinoma, KRAS mutations represent the primary therapeutic target for drug development [27]. Nevertheless, clinical trials targeting KRAS mutations in lung cancer have been disappointing. The failure of these trials may result from the up-regulation of alternative signaling pathways after RAS inhibition or the use of alternative cellular pathways for post-translation modifications of KRAS [28]. Thus, concurrent NRG1 fusion may be a novel therapeutic target for KRAS-mutant IMA.

SLC3A2, also known as CD98hc or 4F2hc, encodes a cell-surface transmembrane protein and was first described as a member of the solute carrier family [29]. SLC3A2 is the heavy chain of a heterodimer. It covalently binds one of several light-chain, L-type amino acid transporters to form heterodimeric neutral amino acid transport systems that contribute to cell survival and growth [30]. Tissues and cell lines representing several cancers including lung cancer have high SLC3A2 expression, which contributes to cell proliferation and adhesion [31, 32]. In concordance, we found that the SLC3A2-NRG1 fusion protein has an SLC3A2 transmembrane domain, the SLC3A2 heavy chain (Figure 1A and 1B). SLC3A2 is part of a cell-surface antigen that contributes to T-lymphocyte activation [19] and has several additional functions such as in thyroid hormone transport and energetic metabolism and modulation of integrin-dependent processes [18]. SLC3A2 establishes tumor cell: SLC3A2-overexpressing NIH-3T3 fibroblasts develop malignant tumors in athymic mice [33]. SLC3A2 overexpression also promotes cell survival and proliferation [34]. When we overexpressed the SLC3A2 gene alone in NSCLC cells, proliferation did not increase (Figure 2A). The SLC3A2 part of the fusion might not have participated directly in the oncogenicity of the fusion gene but might be involved in localization of the fusion protein for paracrine signaling. In addition, transcriptional regulation of SLC3A2 could be important for fusion gene expression. Further study will be needed to investigate these possibilities.

NRG1s are fusion partners of SLC3A2 and are a family of four structurally related proteins (NRG1-4) that are part of the EGF family. NRGs contain an epidermal growth factor (EGF)-like motif that binds and activates receptor-tyrosine kinases in the EGF receptor (ERBBs) family [19, 25, 26]. NRG1 signaling is involved in the development and function of several organ systems [35], human diseases [36], and cancer development [37]. In particular, interaction between ERBB receptors and ligands such as NRGs promotes autophosphorylation of the intracellular tyrosine kinase domain initiating a signaling cascade in tumorigenesis and tumor growth [38]. Although ERBB receptors, except for ERBB2 and ERBB3, can be activated directly after ligand binding and homodimerization, ERBB2 has no soluble high-affinity ligand and ERBB3 has an inactive kinase domain [39–41]. ERBB2 acts mainly as a co-receptor through heterodimerization with the other three receptors and activation *via* ligands. In contrast, ERBB3 serves as a docking protein that is phosphorylated by the other family members. The ERBB2-ERBB3 heterodimer is particularly important for activating the proliferation response of cancer cells [42]. Fusion genes of SLC3A2-NRG1 that are expressed in cancers might be cleaved by metalloproteases. The NRG1 ligands including the EGF-like domain recruit ERBB2-ERBB3 heterodimers that have potent oncogenic signaling that promotes lung cancer growth. Blocking these autocrine or paracrine loops may be an important therapeutic target for controlling cancer cell growth from SLC3A2-NRG1 fusion genes.

In the CD74-NRG1 fusion, the EGF-like domain of NRG1 III-β3 provided ligands to ERBB receptors [24, 25]. In our study, expression of the SLC3A2-NRG1 fusion gene increased cancer cell proliferation *in vitro* and *in vivo* through ERBB2 and ERBB3 heterocomplexes (Figure 2A, 2B, Figure 4, and Figure 5). The EGF-like domain is sufficient for specifically activating ERBB receptors and inducing cellular responses in culture through binding to ERBB receptors and specifically activating ERBB3 and ERBB4 signaling [20]. ERBB3 lacks tyrosine kinase activity and acts only through dimerization with other ERBB components such as ERBB2 [43]. ERBB2 and ERBB3 dimerization triggers the activation of survival and growth signaling cascades, such as through PI3K and MAPK kinases, in both normal and tumor cells [44]. In investigating if the oncogenically functional part of the SLC3A2-NRG1 fusion was the EGF-like domain of NRG1, similar to the CD74-NRG1 fusion, we found that expression of truncated SLC3A2-NRG1Δ EGF counteracted the effect of SLC3A2-NRG1 protein (Figure 2C and 2D). ERBB signaling is emerging as important for lung cancer development [45]. The SLC3A-NRG1 fusion gene we studied also contributed to lung cancer development by inducing oncogenic signaling through ERBB receptors (Figure 5).

Targeting ERBB2 would be an easy therapeutic method using trastuzumab, a monoclonal antibody that interferes with the ERBB2 receptor [46]. Targeting NRG1 is useful because removing the NRG1 domain activates ERBB receptors. Blocking or neutralizing antibodies against NRG1 would be an effective method for preventing the interaction of NRG1 and its receptor. Removing the NRG1 domain from the fusion genes recruited ERBB2-ERBB3 heterocomplexes, resulting in oncogenic signaling that was more potent than signaling from the individual receptors. Pertuzumab, a monoclonal antibody directed against the dimerization arm of ERBB2, delays and impedes NRG1-induced ERBB receptor activation by inhibiting receptor-receptor interaction [47]. The EGF-like domain of the NRG1 protein is regulated by intramembrane proteolytic processing. The NRG1 III-β3 form must undergo this shedding to present an EGF-like domain into the luminal space [48]. The SLC3A2-NRG1 fusion protein included the cleavage site for ADAMs family proteases. The broad spectrum ADAM inhibitor GM6001 might be a therapy for reducing the shedding of the EGF-like domain. Therefore, the fusion gene could have an oncogenic function and serve as a therapeutic target in IMA.

In this study, we analyzed the oncogenic functions and clinical impact of an SLC3A2-NRG1 fusion on IMA. We found that an SLC3A2-NRG1 fusion promoted cancer cell proliferation and migration and tumor volume using a shedding and juxtacrine method through ERBB2-ERBB3 heterocomplexes. The fusion might provide a novel therapeutic target for IMA patients.

## MATERIALS AND METHODS

### Study population

The study included lung adenocarcinoma patients who underwent surgical resection at the National Cancer Center Hospital. Information on sex, age, tumor stage, smoking record, and overall survival was extracted from a clinical database for this cohort. No patients had previous genomic characterization or were enrolled in the Cancer Genome Atlas (TCGA) study of lung adenocarcinoma. The Institutional Review Board of the National Cancer Center Hospital approved this research. All participants provided written informed consent.

### DNA and RNA preparation for next-generation sequencing

Genomic DNA and total RNA were extracted from a single surgical sample (one per patient) that contained primary lung adenocarcinoma and adjacent noncancerous lung tissue. Samples were snap-frozen or stored in RNAlater RNA Stabilization (Qiagen, Germany) solution. Total RNA was isolated using RNeasy Mini Kit columns according to the manufacturer's protocol (Qiagen). RNA quality was determined using the RNA integrity number and was assessed on an Agilent 2100 Bioanalyzer using an RNA6000 Nano Chip (Agilent Technologies, CA, USA). Total RNA quantity was determined using an Infinite 200 PRO NanoQuant Spectrophotometer (TECAN, Switzerland). Genomic DNA was extracted from tissues according to the QIAamp DNA mini kit protocol (Qiagen).

### RNA sequencing *via* next-generation sequencing and fusion transcript detection

Transcriptome libraries were prepared following the Illumina TruSeq RNA sample prep kit protocol using 1-2 μg of total RNA (Illumina, San Diego, CA, USA). Poly(A)^+^RNA was isolated using AMPure XP beads (Beckman Coulter, USA) and fragmented with Ambion Fragmentation Reagents kits (Ambion, Life Technologies, CA, USA). CDNA synthesis, end-repair, A-base addition, and ligation of Illumina indexed adapters were performed according to Illumina protocols. Libraries were size-selected for 250-300 bp cDNA fragments on 3% 3:1 agarose gels. Products were recovered using QIAEX II gel extraction reagents (Qiagen) and PCR-amplified using Phusion DNA polymerase (New England Biolabs, MA, USA) for 14 PCR cycles. The quality of the cDNA library was measured on an Agilent 2100 Bioanalyzer for product size and concentration. Paired-end libraries were sequenced with an Illumina HiSeq 2000, (2 × 100-nucleotide read length). Transcriptome analysis was used the RNAseq Tuxedo protocol [49]. Sequences were mapped against the human reference genome (Ensembl release 69) using TopHat v2.0.9 software (http://tophat.cbcb.umd.edu/) [50] with default options for paired-end sequences and transcript expression estimated using the Cufflinks program v2.1.1 (http://cufflinks.cbcb.umd.edu/) [51]. Fusion-gene discovery was performed using DeFuse v0.5.0 software (http://sourceforge.net/apps/mediawiki/defuse/index.php?title=DeFuse/) [52] and PRADA v1.1 software (http://bioinformatics.mdanderson.org/main/PRADA:Overview) with default parameters. Fusion transcripts with fewer than 5 spanning reads and fewer than 3 split reads were filtered out.

### Fusion-gene verification using reverse transcription polymerase chain reaction and direct sequencing

Known and novel fusion genes were verified using reverse transcription polymerase chain reaction (RT-PCR) followed by Sanger sequencing. RT-PCR of fusion genes used forward and reverse primer pairs in Supplementary Table S3. For reactions, 10 ng cDNA, 400 nM primer, and 0.5 units of HotstarTaq polymerase (Qiagen) were used in 20-μl reactions. RT-PCR was: 15 min at 94°C, 38 cycles at 94°C for 30 sec, 58°C for 30 sec, and 72°C for 1 min, with 5 min at 72°C. PCR products were confirmed *via* direct sequencing using an ABI Prism 3730×l DNA Sequencer (Applied Biosystems) and Big-Dye Terminator ver3.1 Cyclic Sequencing kit (Applied Biosystems, CA, USA).

### Fluorescence *in situ* hybridization analysis

Fluorescence *in situ* hybridization (FISH) was performed with formalin-fixed paraffin-embedded (FFPE) slides. The FFPE samples were prepared at 4μm. Briefly, FFPE tissue sections were deparaffinized, sequentially rehydrated in 100, 85, and 70% ethanol, and incubated in 0.2N HCl for 20min at room temperature. The tissue slides were incubated 8% sodium thiocyanate for 30min at 80°C, followed by treatment with pepsin (0.05% pepsin in 0.01N HCl) for 30min at 37°C. The hydrolysis of tissues were stopped with 1% formaldehyde in PBS, followed by sequential dehydration in 70, 85, and 100% ethanol. Slides were covered with a dual hybridization mixture containing a pair of painting probes (Cy5 and FITC) labeled with MacProbe™ solution (custom generation of probe from Macrogen, Korea). The slides were denatured for 5min at 75°C and hybridized overnight at 37°C in a humidity chamber according to the manufacturer's protocol. The slides were washed and counterstained with 4′,6-diamidino-2-phenylindole (Vector Laboratories Inc.). FISH images were produced using a Leica DMRXA2 (Leica Microsystems, Wetzlar, Germany). Images were captured by a CoolSNAP cf digital camera (Roper Scientific Photometrics, Tucson, USA) and analyzed using a Leica CW4000 (Leica Microsystems, Wetzlar, Germany).

### Detection of cancer hotspot variants using targeted cancer panel deep sequencing

A total of 10 ng DNA was used for multiplex PCR panels covering 739 mutations in 46 key cancer genes: *ABL1, AKT1, ALK, APC, ATM, BRAF, CDH1, CDKN2A, CSF1R, CTNNB1, EGFR, ERBB2, ERBB4, FBXW7, FGFR1, FGFR2, FGFR3, FLT3, GNAS, HNF1A, HRAS, IDH1, JAK2, JAK3, KDR, KIT, KRAS, MET, MLH1, MPL, NOTCH1, NPM1, NRAS, PDGFRA, PIK3CA, PTEN, PTPN11, RB1, RET, SMAD4, SMARCB1, SMO, SRC, STK11, TP53,* and *VHL* (Ion AmpliSeq Cancer Panel, Life Technologies, NY, USA). Fragment libraries were constructed using DNA fragmentation, barcode and adaptor ligation, and library amplification, according to the manufacturer's instructions as specified in Ion DNA Barcoding kits (Life Technologies).

Template preparation, emulsion PCR, and ion sphere particle (ISP) enrichment were performed using Ion P1 Template 200 kits (Life Technologies) according to the manufacturer's instructions. ISPs were loaded onto a P1 chip and sequenced using an Ion P1 sequencing 200 kit on an Ion Proton (Life Technologies). Ion Torrent platform-specific pipeline software Torrent Suite v2.0 and Ion Reporter v4.0 were used to separate barcoded reads, generate sequence alignments with the hg19 human genome reference, perform target-region coverage analysis, and filter and remove poor signal reads. Alignment files in Torrent Suite were transferred to an Ion Reporter for variant file generation using default parameters. For downstream analysis, variants with minimum coverage 500 reads containing at least 20 of the mutant reads were selected. Detected variants were filtered with the in-house normal population variant database KPGP (http://opengenome.net/), followed by selecting variants with variant frequency between 0.05 and 0.4. The last filter step eliminated variants in amplicon AMPL339432 (PIK3CA, exon13, chr3:178938822-178938906), which was not uniquely matched in the human genome. Potential driver mutations identified from deep sequencing were validated using Sanger sequencing.

### Co-culture and culture in conditioned media

Co-cultures were performed in 24-transwell plates (Corning Coster, Cambridge, MA, USA). Cancer cells were seeded in lower chambers and HEK 293T cells were in upper chambers. Cultures in conditioned media were placed in the same plates for co-culture. To obtain conditioned media, HEK 293T cells were transfected and conditioned media filtered (0.45 μM) 3 days after transfection. Conditioned media was transferred to cancer cells after mixing 1:1 with fresh media.

### Cell viability assays

For assays, 2 × 10^3^ cells were plated in 96-well plates under normoxia or hypoxia for 6 days. Cell viability was measured using 3-(4,5-dimethylthiazol-2-yl)-2,5-diphenyltetrazolium bromide (MTT) at 570 nm. Six replicate wells were used per analysis with at least three independent experiments.

### Soft agar assay

To analyze the anchorage-independent cancer growth, cells (2x10^3^/well) were suspended in 0.5% top agar and cultured on 1.0% agar for 4-5 weeks. Cells were stained with 0.05% crystal violet after fixed with 1% paraformaldehyde, and cell masses (> 0.2-mm diameter) were counted as colonies by using ImageJ program.

### Migration assay

The migration assay was performed using an 8.0 μm pore size transwell membrane chamber. Transwell was coated with collagen (0.5 mg/ml, outside). Cells (5x10^4^ cells) in serum-free RPMI medium were loaded into the top chamber with BSA and 10 % FBS contained medium in bottom chamber as a chemoattractant. After incubation at 37 °C for 72 h, the cells on the top of the filters were removed with cotton tips. The cells on the lower surface of the filters were fixed in methanol and stained with 0.1 % crystal violet. The crystal violet was removed and the cells were washed three times with phosphate-buffered saline (PBS). The remaining crystal violet staining of the migrated cells was counted by using ImageJ program.

### Human NRG1-β1 detection assays

Human NRG1-β1 detection assays used human NRG1-β1 ELISA kits (R&D Systems, Minneapolis, MN) with 100 μl cell lysate added to plates prepared according to the manufacturer's procedure and mixed with 100 μl detection antibody with gentle shaking for 2 h at room temperature. Streptavidin-HRP conjugate solution (100 μl) was added and incubated 20 min at room temperature. Substrate solution (100 μl) was added and after 20 min, stop solution (50 μl) was added. Optical density was determined using a microplate reader set to 450 nm.

### Detection of secreted NRG1

Plasmids of SLC3A2, NRG1, SLC3A2-NRG1, or SLC3A2-NRG1Δ EGF (3 μg/μl) were transfected into HEK 293T cells grown in serum-free media at 24 hour after transfection. To concentrate media, collected media were centrifuged with an Amicon ultra filter, and the concentrated media were mixed with 4x sample buffer after protein concentration measurement and then boiled at 95 °C for 8 min. A total of 30 μg of sample was immunoblotted with neuregulin antibody from Thermo Scientific. Coomassie stain was used for loading control.

### Tumor xenografts in mice

All animal procedures were performed in accordance with a protocol approved by National Cancer Center Animal Care and Usage Committee (NCC-14-255). Nude mice (BALB/cAnNCrj-nu/nu) from Charles River Japan Inc. (Shin-Yokohama, Japan) were injected at dorsal flank sites with 1 × 10^6^ cancer cells suspended in 100 μL phosphate-buffered saline. Tumor volume was measured with calipers (volume = L x w x w x 0.52, where L was the width at the widest point of the tumor and w was the width perpendicular to L) when tumors reached a volume of 80-100 mm^3^ (termed day 0 for our experiments). Tumor volume was measured once every 3 days. At the end of experiments, mice were sacrificed by CO_2_ asphyxiation. Excised tumors were cut into two and tissues fixed with 4 % buffered formalin or frozen in liquid nitrogen.

### Statistical analysis

Statistical analyses were performed using the SPSS version 21 (IBM, NY, USA). DFS was defined as time from date of surgery to date of first recurrence. OS was measured from date of surgery to date of death. DFS and OS were evaluated using the Kaplan-Meier method. The log-rank test was used to compare DFS and OS according to NRG1 fusion status. Fisher's exact or chi-square tests were used for determining associations between categorical variables. Results are expressed as mean and standard error of the mean (SEM) or standard deviation (SD) from > 3 independent samples and calculated with Microsoft Excel software 2010. We used unpaired Student's *t*-test for all tests. Differences were considered significant when *P* < 0.05. All statistical tests were two-sided.

## SUPPLEMENTARY FIGURES AND TABLES






