# Supplementary material for: Oncogenic function and clinical implications of SLC3A2-NRG1 fusion in invasive mucinous adenocarcinoma of the lung
Source: Oncotarget. 2016 Sep 8;7(43):69450–65. doi: 10.18632/oncotarget.11913 (PMC5342490; doi:10.18632/oncotarget.11913)
Supplement: Supplementary file 1 [file oncotarget-07-69450-s001.pdf]

Oncogenic function and clinical implications of SLC3A2-NRG1 fusion in invasive mucinous adenocarcinoma of the lung

Supplementary Material

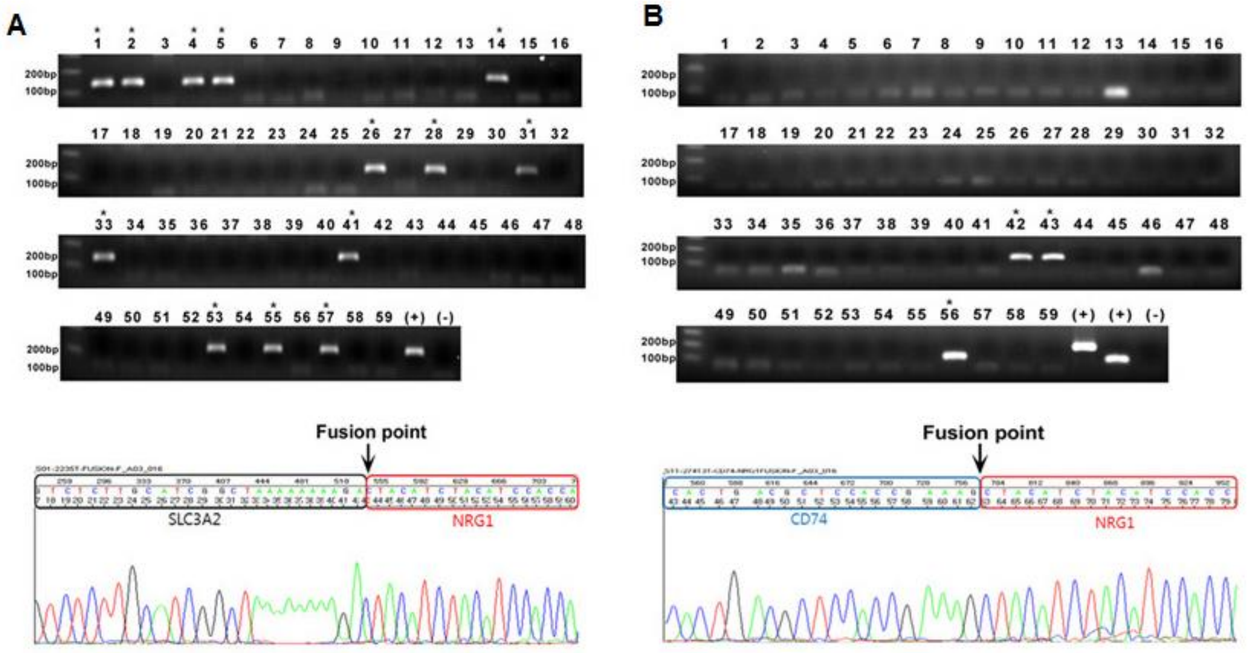

Supplemental Figure 1. Identification of NRG1 fusions in lung mucinous adenocarcinoma by RT-PCR and direct sequencing. Identification of SLC3A2-NRG1 **(A)** and CD74-NRG1 **(B)** fusions in lung mucinous adenocarcinoma by RT-PCR (upper) and direct sequencing (lower).

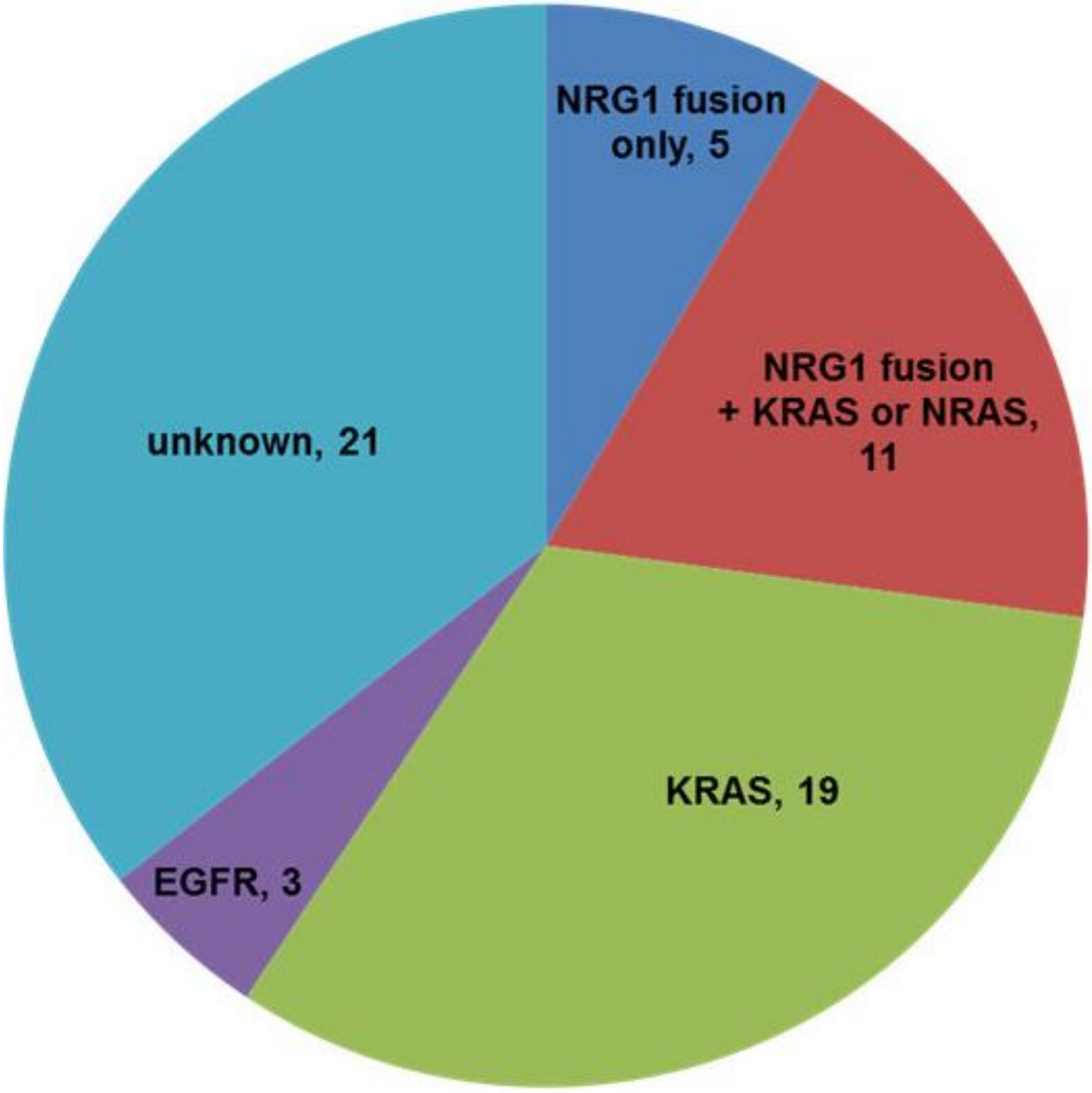

Supplemental Figure 2. Driver mutation found by targeted deep sequencing in lung mucinous adenocarcinoma (total 59 cases).

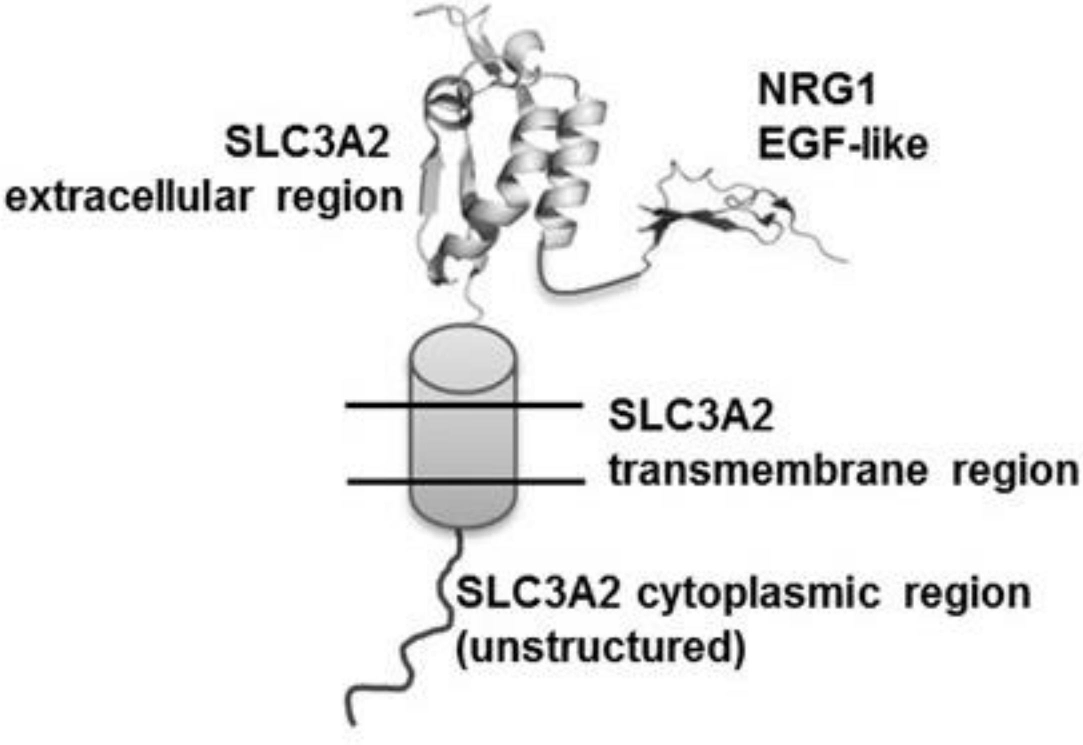

Supplemental Figure 3. Schematic representation of transmembrane domain, fusion junction, and EGF-like domain of SLC3A2-NRG1 fusion gene. TM, transmembrane domain.

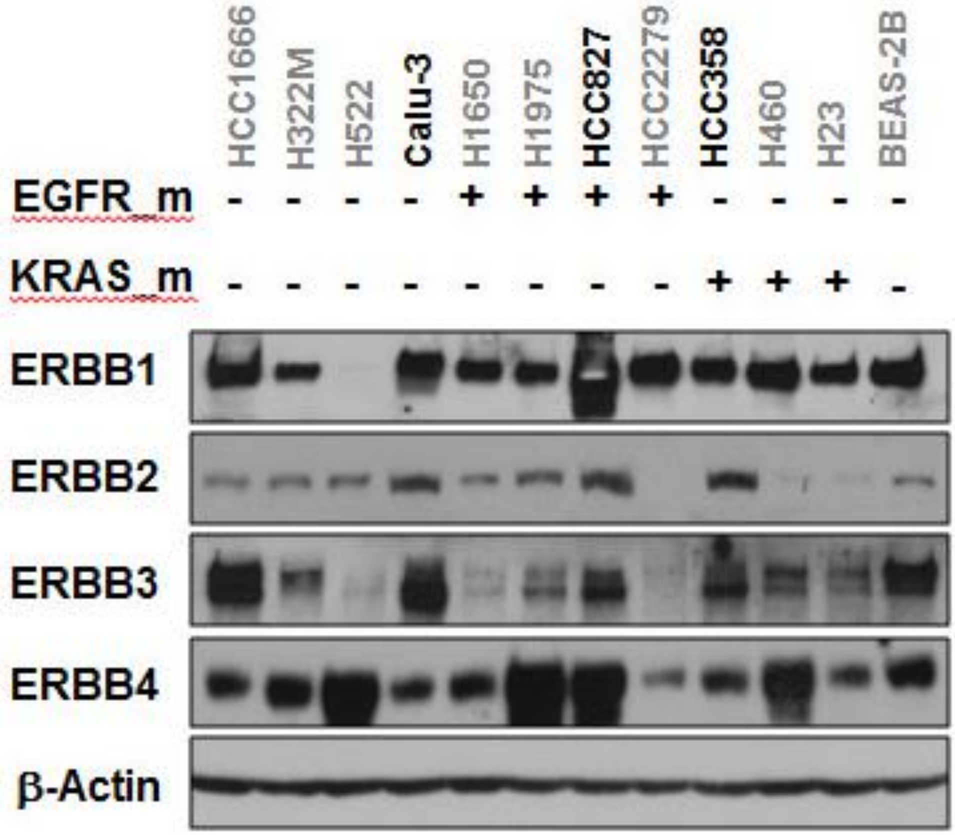

Supplemental Figure 4. ERBB1-4 protein from 11 cancer cell lines and one normal lung epithelial cells (BEAS-2B) by Western blot. β-actin was the loading control.

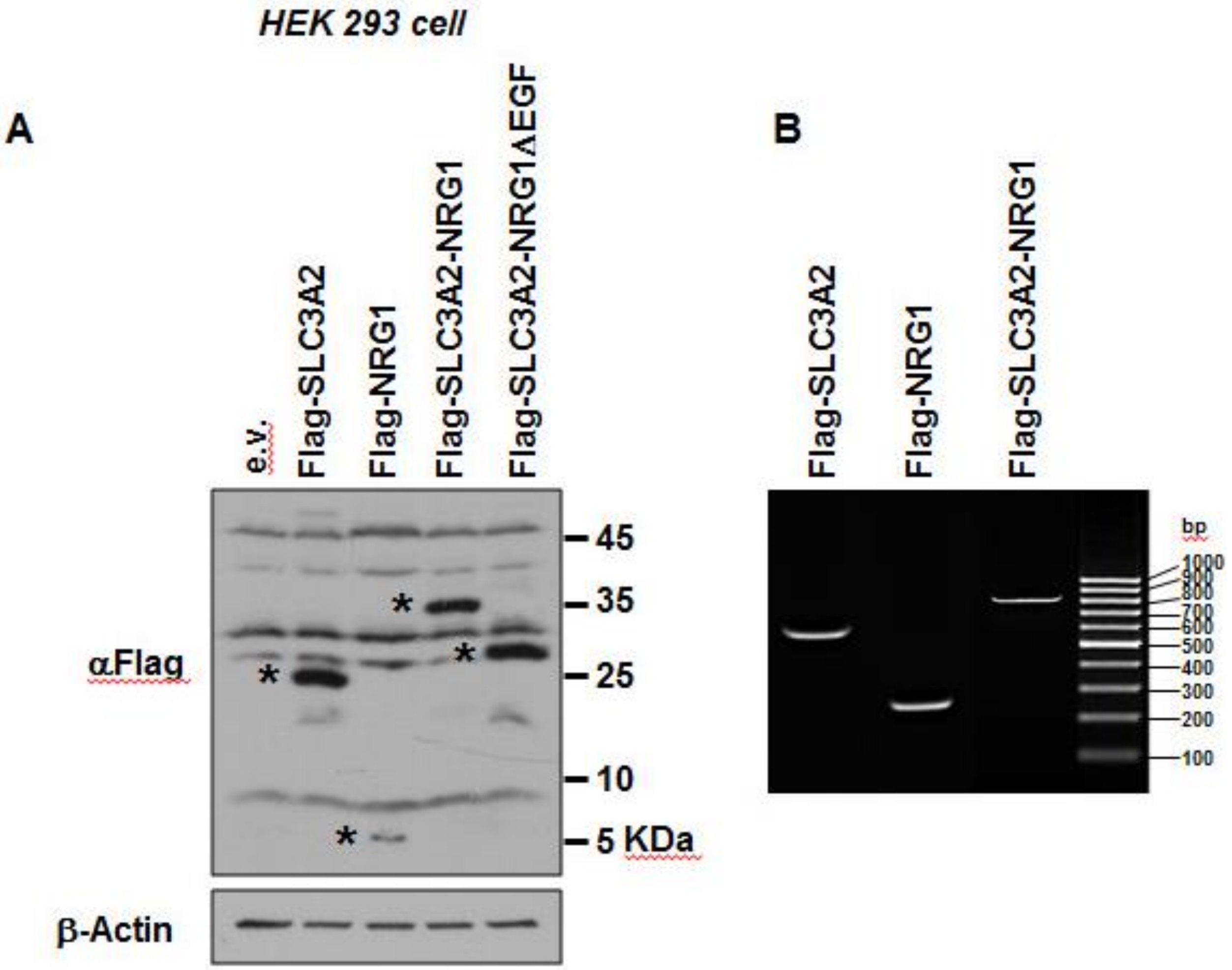

Supplemental Figure 5. Flag-tagged fusion plasmids e.v., SLC3A2, NRG1, SLC3A2-NRG1 and SLC3A2-NRG1ΔEGF transfected and identified by Western blot and PCR.

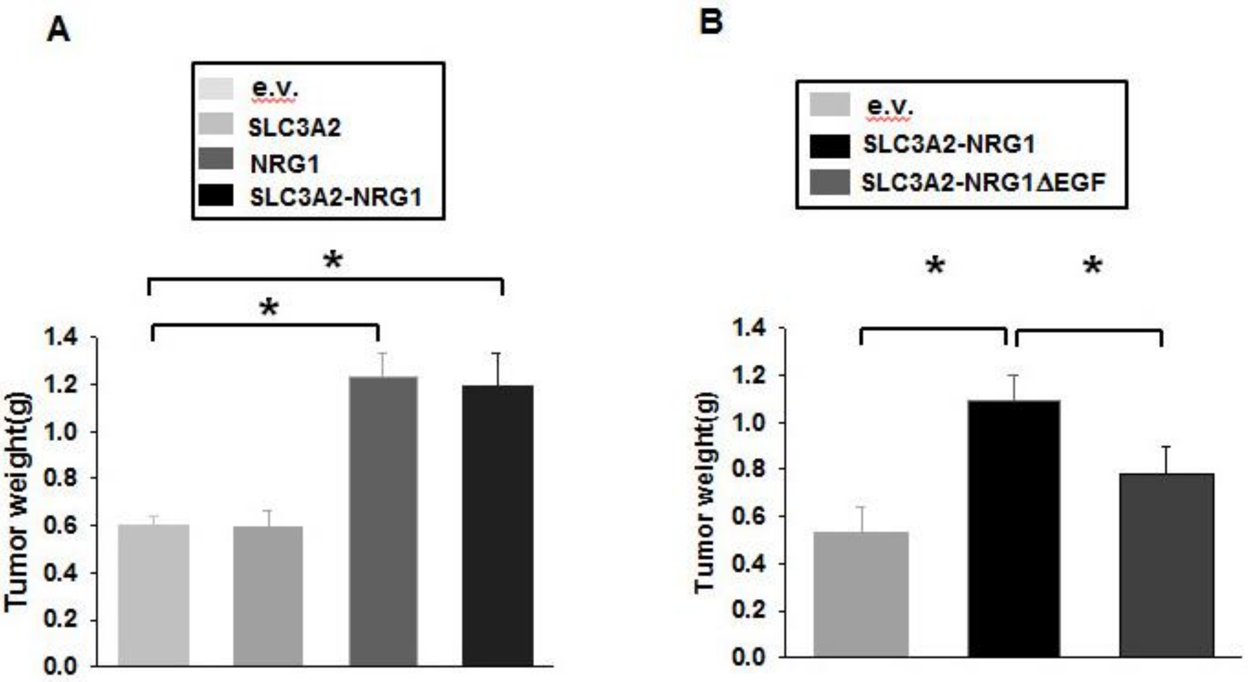

Supplemental Figure 6. Tumor weight from mouse experiments. Bars are average + SEM; n = 5, * $p < 0.05$ by Student's *t*-test.

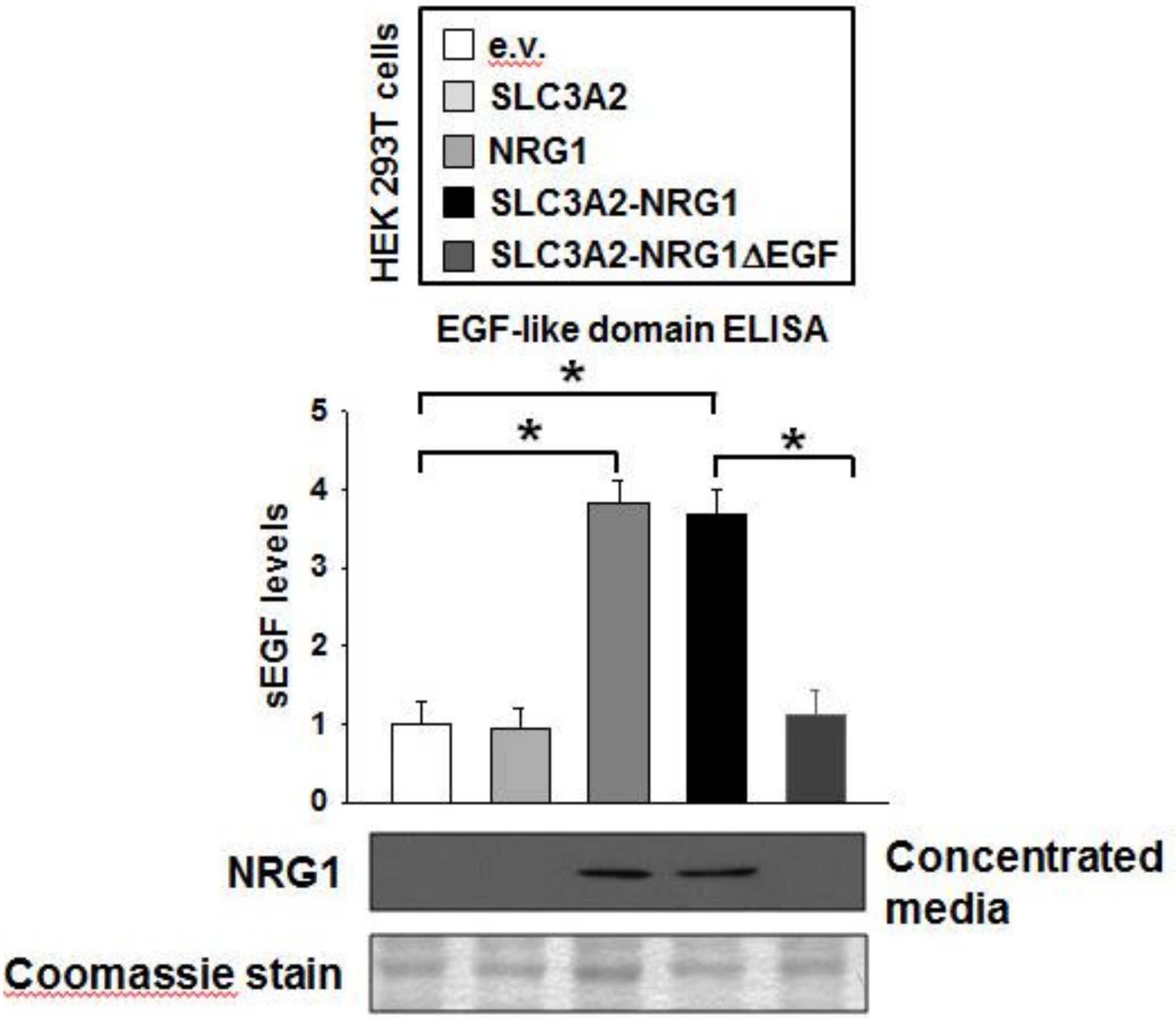

Supplemental Figure 7. Evaluation of secreted NRG1: To evaluate secreted NRG1 part from cells, these plasmids of e.v., SLC3A2, NRG1, SLC3A2-NRG1 and SLC3A2-NRG1ΔEGF (3 μg/μl) transfected in HEK293T cells and changed serum-free media after 24 hour transfection. To condense media concentration, collected media were centrifuged with Amicon ultra filter and measure NRG1 concentration with EGF-like domain ELISA kit. The bars present average + SD; n=6, *, $p<0.05$ by Student's t test (Upper panel). Using the NRG1 antibody in the EGF-like domain (Ab-2, Thermo Scientific, USA), which can detect exogeneous NRG1 fusion products, the secreted NRG1 protein was detected. If exogenous NRG1 fusion products increase the levels of endogeneous native ERBB2/ERBB3 ligands, secreted NRG1 levels (an ERBB3 ligand) in NRG1 single expression (third lane) should be more highly

increased than that in SLC3A2-NRG1 fusion expression (fourth panel). However, secreted NRG1 levels did not show a large difference between the overexpression of NRG1 single and SLC3A2-NRG1 fusion products (fourth lane) (down panel).

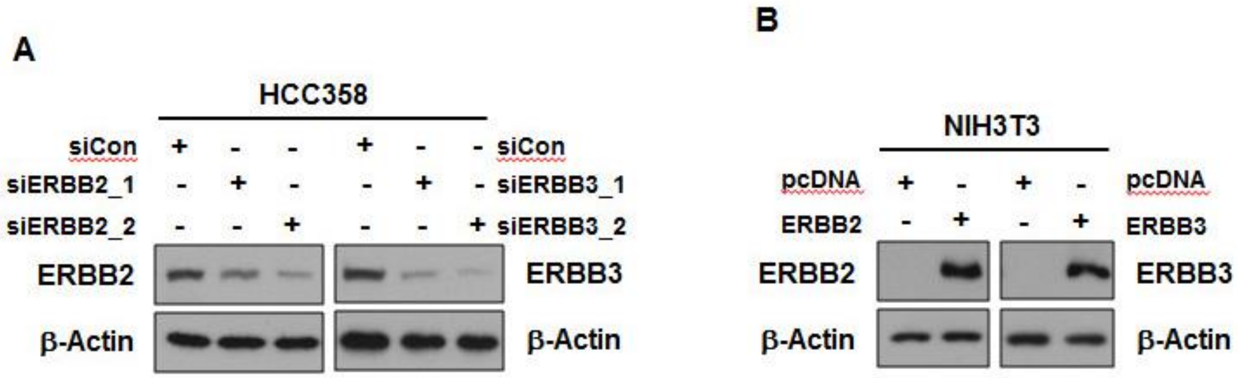

Supplemental Figure 8. HCC358 and NIH3T3 cells were transfected with siERBB2/siERBB3 (80 nM) and ERBB2/ERBB3 (3 μg/μl) and ERBB2 and ERBB3 evaluated by Western blots.

For Supp Table S1, S2, S3, please see the attached word file
