# Supplementary figures and images for: Oncogenic function and clinical implications of SLC3A2-NRG1 fusion in invasive mucinous adenocarcinoma of the lung
Source: Oncotarget. 2016 Sep 8;7(43):69450–65. doi: 10.18632/oncotarget.11913 (PMC5342490; doi:10.18632/oncotarget.11913)

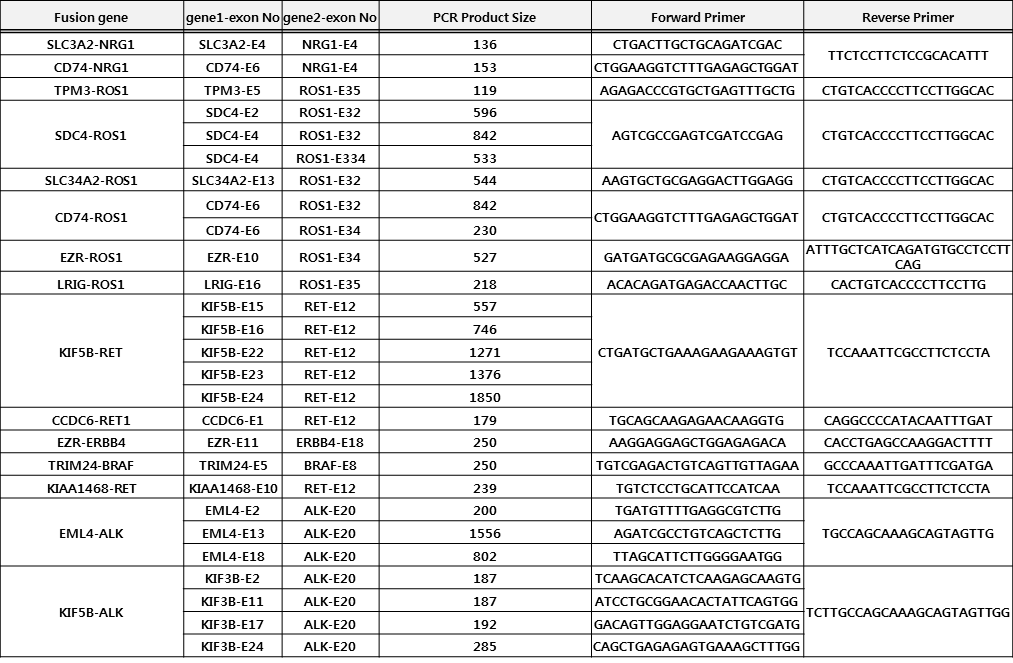


**Supplemental Table 3. RT-PCR primer sets**

Supplement: Supplementary file 3 [file oncotarget-07-69450-s003.docx]
